# Personalized Prediction of Postconcussive Working Memory Decline: A Feasibility Study

**DOI:** 10.3390/jpm12020196

**Published:** 2022-01-31

**Authors:** Yung-Chieh Chen, Yung-Li Chen, Duen-Pang Kuo, Yi-Tien Li, Yung-Hsiao Chiang, Jyh-Jong Chang, Sung-Hui Tseng, Cheng-Yu Chen

**Affiliations:** 1 Translational Imaging Research Center, Taipei Medical University Hospital, Taipei 110, Taiwan; 173002@h.tmu.edu.tw (Y.-C.C.); 194029@h.tmu.edu.tw (D.-P.K.); sandy0932@tmu.edu.tw (C.-Y.C.); 2 Department of Medical Imaging, Taipei Medical University Hospital, Taipei 110, Taiwan; 3 Department of Occupational Therapy, Kaohsiung Medical University, Kaohsiung 807, Taiwan; u107706003@kmu.edu.tw (Y.-L.C.); jjchang@kmu.edu.tw (J.-J.C.); 4 Neuroscience Research Center, Taipei Medical University, Taipei 110, Taiwan; ychiang@tmu.edu.tw; 5 Center for Neurotrauma and Neuroregeneration, Taipei Medical University, Taipei 110, Taiwan; 6 Department of Neurosurgery, Taipei Medical University Hospital, Taipei 110, Taiwan; 7 Department of Surgery, School of Medicine, College of Medicine, Taipei Medical University, Taipei 110, Taiwan; 8 Department of Physical Medicine and Rehabilitation, Taipei Medical University Hospital, Taipei 110, Taiwan; d301091012@tmu.edu.tw; 9 Department of Physical Medicine and Rehabilitation, School of Medicine, College of Medicine, Taipei Medical University, Taipei 110, Taiwan; 10 Department of Radiology, School of Medicine, College of Medicine, Taipei Medical University, Taipei 110, Taiwan; 11 Research Center for Artificial Intelligence in Medicine, Taipei Medical University, Taipei 110, Taiwan

**Keywords:** concussion, mild traumatic brain injury, working memory, long-term cognitive outcome, support vector machine classifier, personalized prediction

## Abstract

Concussion, also known as mild traumatic brain injury (mTBI), commonly causes transient neurocognitive symptoms, but in some cases, it causes cognitive impairment, including working memory (WM) deficit, which can be long-lasting and impede a patient’s return to work. The predictors of long-term cognitive outcomes following mTBI remain unclear, because abnormality is often absent in structural imaging findings. Previous studies have demonstrated that WM functional activity estimated from functional magnetic resonance imaging (fMRI) has a high sensitivity to postconcussion WM deficits and may be used to not only evaluate but guide treatment strategies, especially targeting brain areas involved in postconcussion cognitive decline. The purpose of the study was to determine whether machine learning-based models using fMRI biomarkers and demographic or neuropsychological measures at the baseline could effectively predict the 1-year cognitive outcomes of concussion. We conducted a prospective, observational study of patients with mTBI who were compared with demographically matched healthy controls enrolled between September 2015 and August 2020. Baseline assessments were collected within the first week of injury, and follow-ups were conducted at 6 weeks, 3 months, 6 months, and 1 year. Potential demographic, neuropsychological, and fMRI features were selected according to their significance of correlation with the estimated changes in WM ability. The support vector machine classifier was trained using these potential features and estimated changes in WM between the predefined time periods. Patients demonstrated significant cognitive recovery at the third month, followed by worsened performance after 6 months, which persisted until 1 year after a concussion. Approximately half of the patients experienced prolonged cognitive impairment at the 1-year follow up. Satisfactory predictions were achieved for patients whose WM function did not recover at 3 months (accuracy = 87.5%), 6 months (accuracy = 83.3%), and 1 year (accuracy = 83.3%) and performed worse at the 1-year follow-up compared to the baseline assessment (accuracy = 83.3%). This study demonstrated the feasibility of personalized prediction for long-term postconcussive WM outcomes based on baseline fMRI and demographic features, opening a new avenue for early rehabilitation intervention in selected individuals with possible poor long-term cognitive outcomes.

## 1. Introduction

Mild traumatic brain injury (mTBI), commonly referred to as concussion, typically does not present with visual findings on structural magnetic resonance imaging (MRI) examinations, and therefore, providing neuroimaging evidence to support a diagnosis or therapeutic evaluation is difficult. Furthermore, mTBI can cause an array of postconcussive symptoms (PCS), most notably headaches, sleep deficit, fatigue, dizziness, depression, anxiety, and cognitive impairment [1]. The average time required for symptom relief in most individuals is approximately 3 months [2]; however, some individuals with subjective PCS continue to experience symptoms even 1 year after a concussion [3,4]. Postconcussive neuropsychological deficits have been suggested to be secondary to cognitive deficits [5,6,7]. Studies have suggested that only 15% of first-time concussed individuals continue to experience persistent neuropsychological symptoms [8,9]; however, approximately half of them experience long-term cognitive impairment that persists for years and can severely affect their overall quality of life [10,11]. The options for the early treatment of mTBI remain rather limited due to a general lack of validated biomarkers with a high degree of sensitivity and specificity for the development of symptom-specific therapies. Thus, useful clinical biomarkers for individualized postconcussive management must be urgently identified, particularly to target individuals with poor long-term cognitive outcomes.

Working memory (WM) involves the ability to transiently store and manipulate information to be used for cognitive or behavioral activities. WM deficit is one of the most common postconcussive cognitive impairments [12]. Chen et al. demonstrated reduced activation in the regions of *N*-back WM circuitry in patients with mTBI during both moderate and high WM load conditions compared with healthy controls (HCs), especially prominent under WM 2-back > 1-back conditions [13]. Differences were identified in WM functional activity between both patients with symptomatic mTBI and HCs, as well as patients’ baseline assessments and 6-week follow-ups, whereas no difference was observed in their neuropsychological and behavioral performances, including digit span scores, continuous performance tests, and WM task performances, suggesting that the deficits in WM functional activity estimated from functional magnetic resonance imaging (fMRI) may have a higher sensitivity to mTBI than to neuropsychological and behavioral evaluations alone [13].

We hypothesized that long-term cognitive outcomes of mTBI can better be predicted using pooled fMRI, demographic, and neuropsychological biomarkers than by using neuropsychological evaluations alone. In this prospective observational study, our objectives were to identify fMRI, demographic, or neuropsychological biomarkers at the baseline that could best predict future cognitive changes during the year following a concussion and to construct machine learning-based predictive models to discriminate between patients at a high risk of poor long-term cognitive outcomes and patients with normal recovery. Specifically, the *N*-back WM task (*N* = 1 and 2) was performed to obtain potential disease-related fMRI features, since WM 2-back > 1-back conditions can show the most prominent changes of impaired WM circuitry after mTBI [13]. Additionally, machine learning algorithms can unravel the relationship between input variables (e.g., biomarkers) and response variables (e.g., cognitive outcome) through a data-learning process, which allows for the prediction of future cognitive changes for each individual or the stratification of a patient population based on characteristic features. Furthermore, understanding various potential biomarkers associated with postconcussive WM impairments may render it possible to translate these biomarkers into effective cognitive rehabilitation strategies to improve, or at least mitigate impediments to, the recovery of WM function, which is important in most occupations.

At present, for cognitive rehabilitation management, detailed holistic neuropsychiatric assessments are required to identify, establish, and develop adaptive general or domain-specific interventions, whether adopting a nonpharmacologic or pharmacologic approach [14]; this is particularly true for the treatment of posttraumatic deficits in memory and executive functions, as different compensatory training strategies are applied based on impairment severity [15]. Our aim was to construct a framework for precise individualized predictions of postconcussive cognitive outcomes based on the early fMRI and neuropsychological biomarkers assessed at the baseline to facilitate early therapeutic intervention and individualized rehabilitation strategies. These fMRI predictive biomarkers also exhibit a potential to reflect the functional dynamics of plasticity mechanisms in the injured brain and can be used to evaluate and guide treatment strategies, specifically targeting brain regions involved in postconcussion cognitive decline [13,16,17].

## 2. Materials and Methods

### 2.1. Participants and Neuropsychological Evaluation

Between September 2015 and August 2020, 70 right-handed patients with mTBI and 48 age-, sex-, and education-matched right-handed HCs consented to participate in the study at Taipei Medical University Hospital. Patients were followed up at 6 weeks (*n* = 34; 52.52 ± 6.95 days), 3 months (*n* = 29; 100.96 ± 13.56 days), 6 months (*n* = 28; 195.95 ± 14.61 days), and 1 year (*n* = 25; 376.48 ± 16.52 days) after a concussion. In total, 24 patients (38.6%) completed the baseline and all four follow-up sessions. All participants had normal or corrected-to-normal visual activity and no history of neurological or psychiatric disorders. This study was approved by the Institutional Review Board of Taipei Medical University Hospital before data collection (TMUH TMU-JIRB No. 201504083, N201612008, N201904032, and N202102008) and conducted according to the original and amended Declaration of Helsinki.

The following operational definition of mTBI was used in the current study: patients with closed-head injuries manifesting in a loss of consciousness lasting for <30 min, initial Glasgow Coma Scale score > 13, and normal findings in computed tomography of the entire brain. The exclusion criteria were prior neuropsychiatric illnesses or symptoms, brain injury history, any coexisting or previous neurological illnesses, any medication use that may interfere with the WM ability and/or neuropsychological parameters, and contraindication for MRI. Inclusion criteria for the control group were the same, except for a negative assessment for mTBI and no concussion history.

Neuropsychological assessments, namely six types of clinical symptom measures, the Mini-Mental State Examination (MMSE), and the Wechsler Adult Intelligence Scale, fourth edition (WAIS-IV), were conducted by a clinical psychologist on the same days as the initial and follow-up MRI scans. The six types of clinical symptoms were assessed using the Glasgow Outcome Scale–Extended (GOSE), Pittsburgh Sleep Quality Index, Epworth Sleepiness Scale, Dizziness Handicap Inventory, Rivermead Post Concussion Symptoms Questionnaire (RPQ), Beck Anxiety Inventory, and Beck Depression Inventory, for which higher scores indicate greater symptomatology. The working memory (WM) ability was assessed using the WM index (WMI), arithmetic ability (AMT), and digit span score (DS) in the WAIS-IV test, for which the higher scores indicated better WM ability. Among them, the WMI is derived from the comprehensive performance of WM-related subtests and can be used as a general indicator representing a subject’s WM ability.

### 2.2. MRI Data Acquisition and Experimental Design

MRI data were obtained using a 3T MRI scanner (Siemens MAGNETOM Prisma, Erlangen, Germany), and a 64-channel head coil was used to acquire the fMRI time series. Standard single-shot gradient-echo echo planar imaging-based fMRI (TR/TE = 2000/20 ms, flip angle = 90°, voxel size = 3 × 3 × 3.5 mm^3^, matrix = 64 × 64 × 40, and 105 volumes) was performed. Participants were instructed to keep their eyes closed and not entertain any particular thoughts while remaining awake, alert, and as motionless as possible.

The experimental design of the *N*-back task in fMRI was performed using Presentation software (Version 18.1, Neurobehavioral Systems, Inc., Berkeley, CA, USA) and is presented in Figure S1. An *N*-back task contains three epochs, each composed of a 30-s task period and a 30-s fixation on a crosshair. The interstimulus interval between each trial during a task period was 2 s. In total, 45 trials were performed, with each task consisting of 80% nontarget trials and 20% target trials. In each run of the *N*-back tasks, participants were instructed to pay attention to a series of six-digit numerical stimuli and respond by using the right index finger to press the button whenever the current stimulus matched the number that had been presented *N* times previously (*N* = 1 or 2) [18].

For the co-registration and normalization of fMRI data, three-dimensional T1-weighted magnetization-prepared rapid gradient-echo images (TR/TE/TI = 2300/3.26/1030 ms, flip angle = 8°, voxel size = 1 × 1 × 1 mm^3^, and matrix = 256 × 256 × 176) were obtained.

### 2.3. Data Analysis

#### 2.3.1. fMRI Preprocessing

The anatomical and fMRI data were preprocessed using Statistical Parametric Mapping (SPM12; Wellcome Department, University College London, UK) for slice timing correction, realignment, spatial normalization to MNI space, and spatial smoothing with a 5-mm full-width-at-half-maximum Gaussian kernel. Furthermore, linear and quadratic trends of the fMRI time series were removed.

#### 2.3.2. WM Task Activation and Deactivation Map

To calculate the brain activation and deactivation map during the *N*-back WM task, the experimental paradigm used the convolved canonical hemodynamic response function as the regressor in a general linear model. Six head motion parameters estimated through image realignment by using SPM12 were used as covariates and partially regressed out of the preprocessed fMRI time series. A contrast image corresponding to the main effects of the task performance was created and represented brain activity relative to the implicit baseline of unmodeled variance [19]. Group level activation and deactivation maps were then calculated as a one-sample *t*-test across all participants within each group.

### 2.4. Statistical Analyses

A one-sample *t*-test was used to determine the significance within the HC or mTBI group, and a two-tailed two-sample *t*-test was used to observe between-group differences. A two-tailed paired-sample *t*-test was conducted to examine the significance between the initial and follow-up data. The statistical tests were corrected for multiple comparisons by controlling the false discovery rate (FDR) to *q* = 0.05 to avoid errors related to multiple comparisons in these calculations.

### 2.5. Regions-of-Interest Selection and Percentage Signal Change Calculation

**Figure 1:**
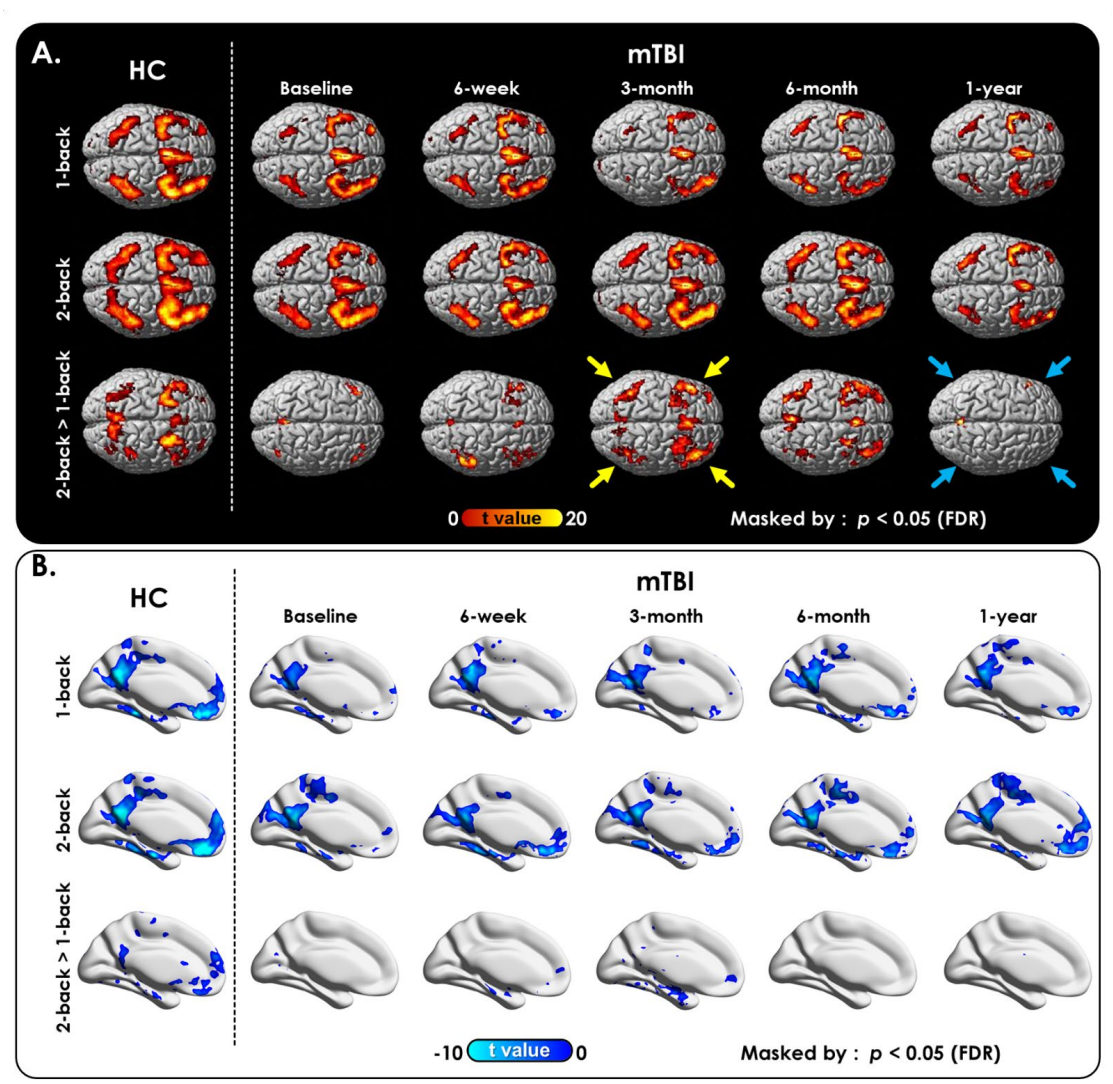
Postconcussive working memory activation and deactivation changes over time between baseline and follow-up. (**A**) Activation and (**B**) deactivation maps of 1-back, 2-back, and 2-back > 1-back WM conditions in HCs and patients with mTBI at each time point. Patients showed significant recovery under the WM 2-back > 1-back condition (bottom row) after 3 months (yellow arrows) but worsened again at the 1-year follow-up (blue arrows). Note that the statistical tests were corrected for multiple comparisons by controlling the false discovery rate (FDR) to *q* = 0.05 to avoid errors related to multiple comparisons in these calculations. Healthy Controls (HCs); mild Traumatic Brain Injury (mTBI).

The regions-of-interest (ROIs; Table S1) were first defined using a 3-mm-diameter sphere centered at the WM 2-back task activation and deactivation peak regions in the HC group (*p* < 0.01, FDR corrected; Figure 1). The percentage signal change map [20] of each participant’s WM condition was then estimated through multiplication of the regression coefficient map for the main effects of task performance approximated as the quotient by dividing the peak value by the constant term in the design matrix such that they could be compared across participants [19]. Finally, the percentage signal change value of each participant’s WM condition at each ROI was then extracted.

### 2.6. Postconcussive WM Changes at Predetermined Time Periods during 1-Year Follow-Up

To determine the courses of the postconcussive WM changes, we first retrospectively assessed the 24 patients who completed the baseline and all four follow-up sessions to determine the status of WM decline or recovery at each time point during the 1-year follow-up period. A two-tailed paired-sample *t*-test was applied to examine the significance between the baseline and follow-up data to identify the statistically significant progression of postconcussive cognitive decline at predetermined time points (periods). Specifically, these statistically significant time periods of cognitive changes were regarded as the meaningful time periods for the machine learning-based approach in terms of postconcussive cognitive progression prediction. Patients were further divided into “poor outcome” and “good outcome” groups according to the negative and positive slopes of cognitive changes within the specific time period.

### 2.7. Individualized Prediction of Postconcussive WM Impairments by Using Biomarkers Measured at Baseline

The percentage signal change extracted from the ROIs for the WM task performed by each participant was used for the *N*-back WM fMRI features. Neuropsychological assessments (namely seven types of neuropsychological tests, clinical symptom measures, and the WAIS-IV test) and the demographic data (namely age, sex, education year, and score on the GOSE) were treated as the potential neuropsychological and demographic features, respectively. The candidate fMRI, demographic, and neuropsychological features were selected if there was a significant correlation with the estimated changes in the WMI during the specific time period to train the support vector machine (SVM) classifier with k-fold cross-validation (k = 10 in this study) for each prediction to achieve a reliable and unbiased estimate of the machine learning model performance on a limited dataset sample [21]. Specifically, the complete dataset is first divided into k consecutive folds. Then each fold is used once as a validation, and the remaining k−1 folds form the training set. This approach may be computationally expensive, but it does not waste too much data (as is the case when fixing an arbitrary validation set), which is the main advantage in problems with very small sample sizes. The individual SVM classification approach was accomplished using in-house MATLAB (version R2020a, MathWorks, Sherborn, MA, USA) scripts.

## 3. Results

### 3.1. Demographics

In total, 70 patients with mTBI (age = 37.9 ± 12.2 years; 23 (32.9%) women) and 48 HCs (age = 37.4 ± 12.0 years; 16 (33.3%) women) were recruited in this study (Figure S2). The reasons for injury were as follows: motor vehicle accident (*n* = 37), fall (*n* = 17), sports (*n* = 3), assault (*n* = 8), and other (*n* = 5). Among the patients, 24 completed the baseline, and all four follow-up sessions were selected for investigations regarding dynamic changes in cognitive functions after a concussion. The patients dropped out during follow-up visits mainly because of a failure to keep in touch through phone calls or e-mails or a change in residence or job. Table S2 lists the basic demographic characteristics of both groups. No significant between-group differences in terms of age, sex, and education were observed between the patients and HCs. Furthermore, no significant within-group differences were observed in the demographics between patients who completed the 1-year follow-up and those who completed only the baseline assessment following concussion. All structural MRI were unremarkable in terms of structural or signal changes.

### 3.2. Postconcussive WM Changes during the 1-Year Follow-Up Period

#### 3.2.1. N-Back WM Task

In both groups, the *N*-back WM task fMRI exhibited increased signals in the bilateral frontal and parietal lobes (*p* < 0.01, FDR-corrected; Figure 1A), consistent with the activation of WM circuitry. However, the extent of activation was less in the patient group than in the HCs (Figure 1A, first two columns). Furthermore, the response of the brain to the increase in WM load from 1-back to 2-back, as shown in the brain activation (Figure 1A, bottom row) and deactivation (Figure 1B) maps, was greater in the HCs than in patients with mTBI. The WM deficit pattern of WM 2-back > 1-back and activation regions were recovered at the third month of follow-up; however, the WM deficit (decline) paradoxically reappeared again at 1-year follow-up after a concussion. Lastly, deactivation in the patient group constantly disappeared at the WM 2-back > 1-back activation regions throughout the 1-year follow-up. In addition, as the WM load increased from 1-back to 2-back, there was an imbalance in the communication between task-positive (activation) and task-negative (deactivation) regions in the context of effortful task execution. Collectively, these findings revealed changes that persist during the chronic phase of mTBI and highlight the need for longitudinal studies to map the postconcussive cognitive decline and/or recovery.

#### 3.2.2. Neuropsychological Assessment

**Figure 2:**
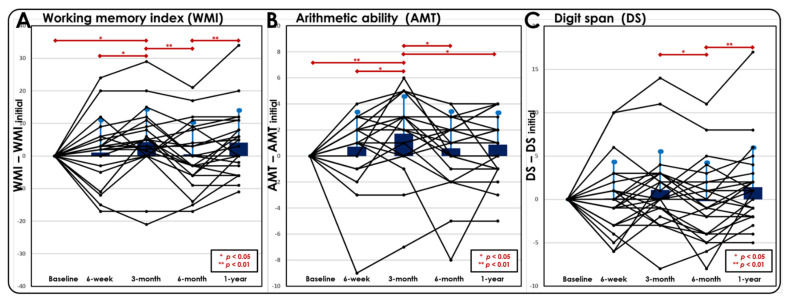
Postconcussive cognitive changes over time between the baseline and follow-up. Dynamic individual patient trajectories of (**A**) the WMI, (**B**) AMT, and (**C**) DS at each time point. The trajectories were normalized by subtracting the baseline measurements for better visualization. Patients exhibited significant recovery during the 3-month follow-up but worsened again from 3 months to 6 months or even at the 1-year follow-up. Compared with the baseline measurements, roughly half of the patients with a mTBI displayed reduced cognitive function after 1 year. (* *p* < 0.05, ** *p* < 0.01).

An assessment with the WAIS neuropsychological test revealed that the WM-related abilities, as indicated by the WMI (Figure 2A), AMT (Figure 2B), and DS score (Figure 2C), significantly improved from 6 weeks to 3 months after the mTBI but became worse again from 3 to 6 months after the mTBI. This result matched with our fMRI results and provided converging evidence that patients tend to have a transient cognitive recovery at the third month after a mTBI; however, it worsens again after 6 months. Since the WMI is derived from the comprehensive performance of WM-related subtests, it is treated as a general indicator representing a subject’s WM ability in our study. Due to the fluctuation and variation in the functional recovery of WM among patients, we grouped patients according to follow-up time periods, where the patients with recovered WMI were classified into the “good outcome group” and the patients exhibiting a decline in WMI were labeled as the “poor outcome group” for the prediction model analysis. The percentages of patients in the four predefined time periods were as follows:

1. Thirty-eight percent (9/24) of patients exhibited no recovery in the WMI at 3 months after a mTBI.
2. Seventy-five percent (18/24) of patients exhibited a decline in the WMI from 3 to 6 months after a mTBI.
3. Thirty-eight percent (9/24) of patients exhibited no recovery in the WMI from 6 months to 1 year after a mTBI.
4. Forty-six percent (11/24) of patients exhibited a worsened WMI at 1-year follow-up compared to the baseline.

### 3.3. Prediction of Postconcussive WMI Decline Based on Baseline Studies

#### 3.3.1. WMI Not Recovered at 3 Months after mTBI

**Figure 3:**
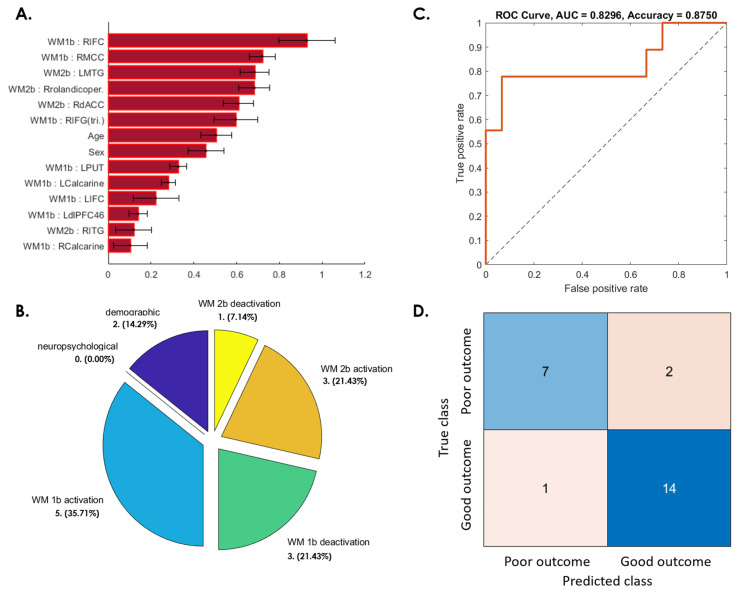
SVM predictive model for 37.5% of patients whose WM ability did not recover at the 3-month follow-up. (**A**) The red bar graph and the corresponding error bar, respectively, represent the average and standard deviation of the discriminative feature weights among the 10 cross-validated SVM classifiers. (**B**) Profiles of selected features for constructing the SVM classification model. None of the neuropsychological features were selected for this predictive model. (**C**) ROC curve of the selected feature to differentiate the “poor outcome group” from the “good outcome group”. (**D**) Confusion matrix to summarize the results of this binary classification model.

Figure 3A presents the important weighting of features derived from candidate fMRI activation and deactivation patterns, demographics, and neuropsychological tests for predicting WMI changes between 6 weeks and 3 months after a concussion. In particular, the selected features included two demographic (age and sex); five WM 1-back activation (left putamen, bilateral calcarine, left dorsolateral prefrontal cortex (dLPFC; BA46), and the triangular part of the right inferior frontal gyrus (IFG (tri.); BA45)); three WM 1-back deactivation (e.g., right middle cingulate cortex (MCC)); three WM 2-back activation (right dorsal anterior cingulate cortex (dACC), right Rolandic operculum, and right inferior temporal gyrus (ITG)); and one WM 2-back deactivation (the left middle temporal gyrus (MTG)) features (Figure 3A,B); 87.5% SVM prediction accuracy and an 82.96% area under the receiver operating characteristic curve (ROC-AUC; Figure 3C,D) were achieved using these features.

#### 3.3.2. WMI Decline from 3 to 6 Months after Initial Recovery

**Figure 4:**
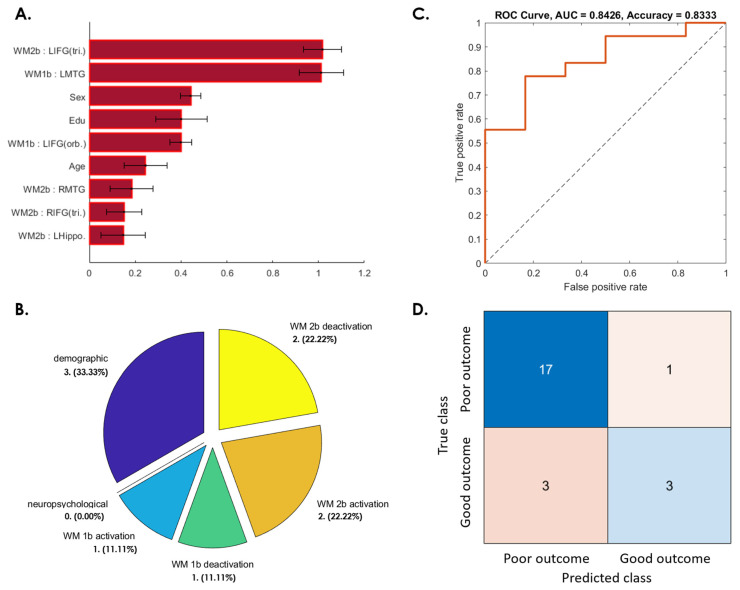
SVM predictive model for 75% of patients whose WM ability dropped from 3 to 6 months after a concussion. (**A**) The red bar graph and the corresponding error bar, respectively, represent the average and standard deviation of the discriminative feature weights among the 10 cross-validated SVM classifiers. (**B**) Profiles of the selected features for constructing the SVM classification model. None of the neuropsychological features were selected for this predictive model. (**C**) ROC curve of the selected feature to discriminate the “poor outcome group” from the “good outcome group”. (**D**) Confusion matrix to summarize the results of this binary classification model.

Figure 4A presents the important weighting of features selected from among the candidate fMRI results, demographics, and neuropsychological tests to predict the WMI changes between 3 and 6 months after a concussion. These features included three demographic (age, sex, and education years); one WM 1-back activation (left inferior frontal gyrus orbital part (IFG (orb.); BA46)); one WM 1-back deactivation (left MTG), two WM 2-back activation (e.g., bilateral IFG (tri.)); and two WM 2-back deactivation (left hippocampus and right MTG) features (Figure 4A,B). The SVM prediction accuracy could reach 83.33%, with an 84.26% ROC-AUC (Figure 4C,D).

#### 3.3.3. WMI Not Recovered from 6 Months to 1 Year after mTBI

**Figure 5:**
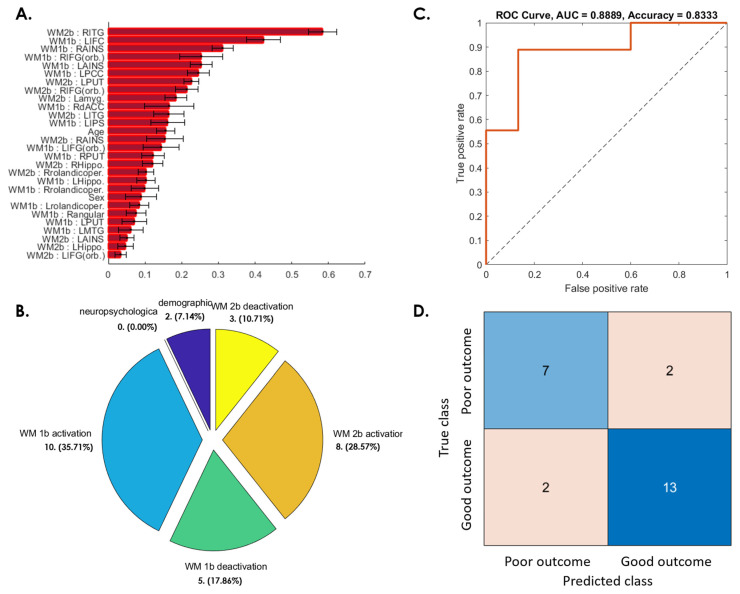
SVM predictive model for 37.5% of patients whose WM ability did not recover from the 6-month to 1-year follow-up. (**A**) The red bar graph and the corresponding error bar, respectively, represent the average and standard deviation of the discriminative feature weights among the 10 cross-validated SVM classifiers. (**B**) Profiles of the selected features for constructing the SVM classification model. None of the neuropsychological features were selected for this predictive model. (**C**) ROC curve of the selected feature to discriminate the “poor outcome group” from the “good outcome group”. (**D**) Confusion matrix to summarize the results of this binary classification model.

Figure 5A shows the important weighting of features selected from candidate fMRI results, demographics, and neuropsychological tests for predicting the WMI changes between 6 months and 1 year after a concussion. The features selected for SVM classification included 2 demographic features (age and sex); 10 WM 1-back activation features (bilateral anterior insula (AINS), bilateral IFG (orb.), bilateral putamen, bilateral Rolandic operculum, right dACC, and left inferior parietal sulcus (IPS)); 5 WM 1-back deactivation features (left posterior cingulate cortex (PCC), right angular gyrus, left hippocampus, left MTG, and left inferior frontal cortex (IFC)); 8 WM 2-back activation (bilateral IFG (orb.), bilateral AINS, bilateral ITG, left putamen, and right Rolandic operculum); and 3 WM 2-back deactivation features (bilateral hippocampus and left amygdala) (Figure 5A,B). With the use of the selected features, the SVM classifier could reach 83.33% accuracy, with an 88.89% ROC-AUC (Figure 5C,D).

#### 3.3.4. Patients Whose WMI at 1-Year Follow-Up Was Worse Than at Baseline

**Figure 6:**
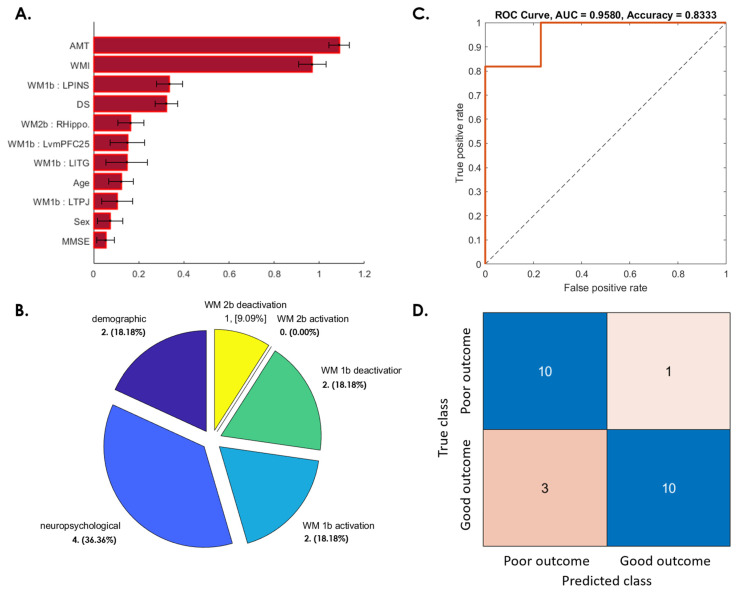
SVM predictive model for 45.83% of patients whose WM ability after 1 year became worse than at the baseline. (**A**) The red bar graph and the corresponding error bar, respectively, represent the average and standard deviation of the discriminative feature weights among the 10 cross-validated SVM classifiers. (**B**) Profiles of the selected features for constructing the SVM classification model. None of the WM 2-back activation features were selected for this predictive model. (**C**) ROC curve of the selected feature to discriminate the “poor outcome group” from the “good outcome group”. (**D**) Confusion matrix to summarize the results of this binary classification model.

The importance of the feature weighting derived from the candidate fMRI, demographic, and neuropsychological features to predict the estimated changes of the WMI between the baseline and 1-year after a concussion is shown in Figure 6. Two demographic features (age and sex); four neuropsychological features (MMSE, DS, WMI, and AMT); two WM 1-back activation features (e.g., left ITG and left temporal parietal junction (TPJ)); two WM 1-back deactivation features (e.g., left ventromedial prefrontal cortex (vmPFC; BA25)); left posterior insula (PINS); and one WM 2-back deactivation feature (e.g., right hippocampus) were selected (Figure 6A,B), and the SVM reached 83.33% accuracy, with a 95.80% ROC-AUC (Figure 6C,D).

## 4. Discussion

This study examined postconcussion cognitive changes during a 1-year follow-up period. Consistent with a previous study [22], in our study, patients with mTBI demonstrated significant cognitive recovery at the third month after a concussion, followed by a worsened performance after 6 months, which persisted until 1 year after a concussion (Figure 1). Approximately half of the patients experienced prolonged cognitive impairment, including impaired WM, DS, and AMT, at the 1-year follow up (Figure 2). The results are similar to those of previous studies, indicating that significant postconcussion cognitive impairment may persist for years, despite some recovery over time [10,11]. Even a single concussion can lead to persistent cognitive impairment in approximately half of patients [10]. We constructed machine learning-based predictive models to differentiate patients at high risk for poor cognitive outcomes at the representative time periods after a concussion. Satisfactory predictions were achieved for patients with mTBI whose cognitive function did not recover after 3 months (Figure 3), worsened at 6 months (Figure 4), did not recover at 1 year (Figure 5), and worsened at 1 year compared with the baseline (Figure 6). This study demonstrated the feasibility of prediction individualization for long-term postconcussion cognitive outcomes by using pooled fMRI, demographic, and neuropsychological features and further suggests the possibility of early therapeutic intervention, such as neurocognitive training, for individuals with mTBI with poor long-term cognitive outcomes to reduce postconcussive cognitive decline and the risk of chronicity [23]. fMRI may also be used to evaluate and guide treatment strategies, specifically targeting brain areas involved in postconcussive cognitive decline [13,16,17].

### 4.1. Validate Machine Learning Algorithms in a Limited Data Size

Previous studies that aimed at predicting long-term postconcussive cognitive outcomes for mTBI generally adopted a multivariate approach encompassing patient demographics, clinical symptoms, and neuropsychological features, as well as other factors such as health care utilization and premorbid psychiatric conditions [24,25]. However, the performance of such predictive methods can be limited due to clinical variability and complexity, as well as confounding factors such as ambiguous documentation, undeclared medication use, and other concurrent medical conditions, and the assessment of morphologic information based on structural brain imaging has not demonstrated additional benefits [26,27]. In this observational study, we prospectively recruited 70 patients with mTBI and followed up their cognitive functioning with functional and neuropsychological data for 1 year. In particular, 24 patients who completed all the baseline and 5 follow-up sessions were selected for retrospective determination of the status of WM decline or recovery at each time point during the 1-year follow-up period. Although the relatively small dataset used in this study might be a concern, k-fold cross-validation (k = 10 in our case) was applied to generate a reliable and unbiased estimate of machine learning model performance on a limited dataset sample [21]. Stated otherwise, we used a limited sample to estimate how the model is expected to perform in general when used to make predictions on unseen data. The systematic processing procedures and the results of this study thus proved the feasibility of using machine learning-based approaches to reveal predictive biomarkers related to poor postconcussive WM outcomes.

### 4.2. Neuropsychological Assessments Are Not Predictive of Postconcussion Cognitive Decline

None of the baseline assessments of clinical neuropsychiatric symptoms (e.g., GOSE, sleep quality, depression, and anxiety) or the self-reported PCS burden (RPQ) correlated significantly with the WM changes between 6 weeks and 3 months, between 3 and 6 months, and between 6 months and 1 year after a concussion in this study. These results substantiate the idea that the baseline neuropsychological assessments and PCS burden may not be predictive of postconcussive cognitive outcomes. Moreover, this was true in a previous study, which demonstrated that a lower cognitive reserve, but not a worse PCS diagnosis, was associated with a poor cognitive outcome following mTBI [28]. Studies have also shown that mTBI-induced differences in WM functional activity are observable; however, differences in the neuropsychological and behavioral performances were not evident, suggesting that the deficits of WM functional activity estimated from fMRI may have a higher sensitivity to long-term WM deficits in mTBI than to neuropsychological evaluations alone [13]. In addition, the time period between a concussion and baseline assessment is also not related to the postconcussive WM changes, which indicates that the biomarkers identified in this study were not biased by the starting time of the initial scan (within 1 week after mTBI in this research). Collectively, these results provide supportive evidence for using fMRI biomarkers elicited from baseline WM functional activity to predict long-term postconcussive cognitive outcomes, as done in our research.

### 4.3. Age and Sex Effects in Postconcussive Working Memory Impairment

Recent studies have indicated the importance of age and sex effects in the context of mTBI, as the elderly and females are especially predisposed to postconcussive neurocognitive symptoms [29,30,31,32]. This is also true in our data, where the age and sex factors showed significant contributions to the predictive models (Figure 3, Figure 4, Figure 5 and Figure 6), which possibly points to the age and sexual vulnerability factors in persistent postconcussive WM impairments. Further investigation is needed to corroborate the findings and to identify the mechanisms behind the involvement of age and sex in mTBI, especially in long-term postconcussive WM outcomes.

### 4.4. The Role of WM Task-Induced Deactivation Regions in Reflecting Postconcussive Cognitive Decline

In addition to considering WM functional activity as a potential fMRI biomarker for postconcussive WM decline, the WM task-induced default mode network (DMN) deactivation regions were considered in this research. The DMN, which is known to be active during rest and to deactivate during externally oriented tasks, may be essential for optimal WM operation [33]. The failure to deactivate the DMN during a cognitive task may limit the ability to reallocate cognitive resources for task execution [34,35]. A superior WM performance might be associated with the balance in communication between task-positive (activation) and task-negative (deactivation) regions in the context of effortful task execution [36]. Therefore, exclusively examining the abnormalities in aberrant activation may be insufficient for a complete understanding of WM pathology [33]. Thus, we focused on both activation and deactivation deficits, marking a substantive advancement over prior works. In our work, approximately one-third to one-half of the fMRI biomarkers exhibited deactivation deficits and significantly contributed to the prediction of postconcussive WM impairments. The results suggest that characterizing both activation and deactivation deficits is crucial for a complete understanding of WM dysfunction in mTBI.

### 4.5. Scientific Merit and Clinical Implications

The systematic characterization of WM functional deficits may have crucial therapeutic implications in patients with postconcussive WM dysfunction, facilitating rehabilitation intervention planning in selected patients. Studies have suggested the potential application of brain functional activation and deactivation patterns in WM tasks for early neurocognitive training referral, training intensity planning, or even functional recovery prediction [13,37]. Moreover, pharmacological interventions such as catecholaminergic treatment with methylphenidate improve the cognitive performance in patients with severe TBI through the normalization of WM activation patterns [38]. Manktelow et al. demonstrated that compromised functional integrity and connectivity strength between key structures of the WM activation pattern in patients with TBI can be treated with methylphenidate to improve cognitive performance and that methylphenidate’s pharmacologic effect may be more beneficial in patients with moderately severe cognitive deficits [39]. This treatment outcome correlation has not been well-explored for mTBI; nevertheless, our study results also indicate that the assessment of WM functional deficits may facilitate early rehabilitation interventions for patients with possible poor long-term cognitive performances and may thus reduce the heterogeneity in treatment responses and cognitive outcomes. These fMRI predictive biomarkers exhibit the potential to reflect the functional dynamics of the neuroplasticity mechanisms in an injured brain. Our results support the hypothesis that pooled fMRI, demographic, and neuropsychological baseline biomarkers can satisfactorily predict postconcussive WM deficits during a 1-year period. Future studies must focus on using these predictive biomarkers as a patient stratification strategy to provide early intervention for patients who are at a high risk of postconcussive WM dysfunction.

### 4.6. Limitations

#### 4.6.1. Small Data Size and Dropouts in Longitudinal Data

This study longitudinally tracked postconcussion WM functions in 70 patients and delineated the trends of dynamic changes in WM ability during a 1-year follow-up period. However, we note the considerable number of dropouts in our longitudinal data, which may be indicative of attrition bias, meaning that perhaps only patients who still felt uncomfortable at the designated follow-up time points would be motivated to continue participating in this study. Therefore, whether the dynamic changes in postconcussion WM functions in this study are representative of the patient population remains somewhat controversial. To mitigate the issue of attrition bias, only 24 patients who completed the baseline and all four follow-up sessions were selected for investigations regarding dynamic cognitive changes in the 1-year period. In fact, no significant within-group differences were observed in the demographics between patients who completed or did not complete the five visits during the 1-year period. In addition, our study showed similar trends of postconcussion cognitive changes with previous studies showing significant cognitive recovery within three months [22], and approximately half of the patients experienced persistent postconcussion cognitive impairments over years [10,11]. Taken together, we can confirm that the 24 patients who completed all five visits in our study were roughly representative of the patient population or at least not significantly different from the patient population. A further study with more longitudinal data is needed to clarify this issue. 

#### 4.6.2. The Handedness and Brain Lateralization

In this study, only right-handed patients and HCs were recruited in this study. Whether left-handed brains are structurally different from right-handed brains is still controversial and has spurred much research. Handedness is a form of functional hemispheric asymmetries that establish differences between the left and right sides of the brain [40]. For the handedness issue, it is not possible to fit the prediction models to left-handers just by simply mirroring left and right brains in the fMRI results. Studies have shown that, in left-handed people, both sides of the brain tend to communicate more effectively, and they have less lateralized brains [40]. Left-handers may also differ in postconcussion functional compensation of the brain network after a mTBI. Further research on the mechanisms of postconcussion WM function changes specifically for left-handed people is needed to clarify this issue.

## 5. Conclusions

This study depicts a framework for the precise individualized prediction of postconcussion cognitive outcomes based on demographic features and early fMRI biomarkers assessed at the baseline to fulfill the demands of early therapeutic intervention and individualized cognitive rehabilitation strategies. fMRI can be a useful tool in revealing the impairment and compensation of the brain cognitive network, which has the potential to guide treatment strategies, specifically targeting the brain areas involved in postconcussion cognitive decline at the individual level. Future prospective studies using this machine learning-based patient stratification strategy may refine and validate the prediction models and further facilitate the clinical applications of postconcussion cognitive decline.

## Data Availability

Open-source software is available from the resources as cited or from the authors on request. Other source codes generated in this study can be downloaded from https://github.com/YiTienLi/matlab-toolbox (accessed on 31 December 2021). The data are not publicly available due to the wording of the consent that participants gave to this project.

## Supplementary Materials

The following are available online at www.mdpi.com/article/10.3390/jpm12020196/s1: Figure S1: *N*-back WM task diagram. Figure S2: Flow chart. Table S1: WM 2-back task-induced activation and deactivation peak regions among all participants. Table S2: Demographics, clinical, and cognitive characteristics and WM task performance of the participants.
