# Supplementary material for: Personalized Prediction of Postconcussive Working Memory Decline: A Feasibility Study
Source: J Pers Med. 2022 Jan 31;12(2):196. doi: 10.3390/jpm12020196 (PMC8878610; doi:10.3390/jpm12020196)
Supplement: Supplementary file 1 [file jpm-12-00196-s001.zip › jpm-1532882-supplementary.pdf]

Supplementary Materials

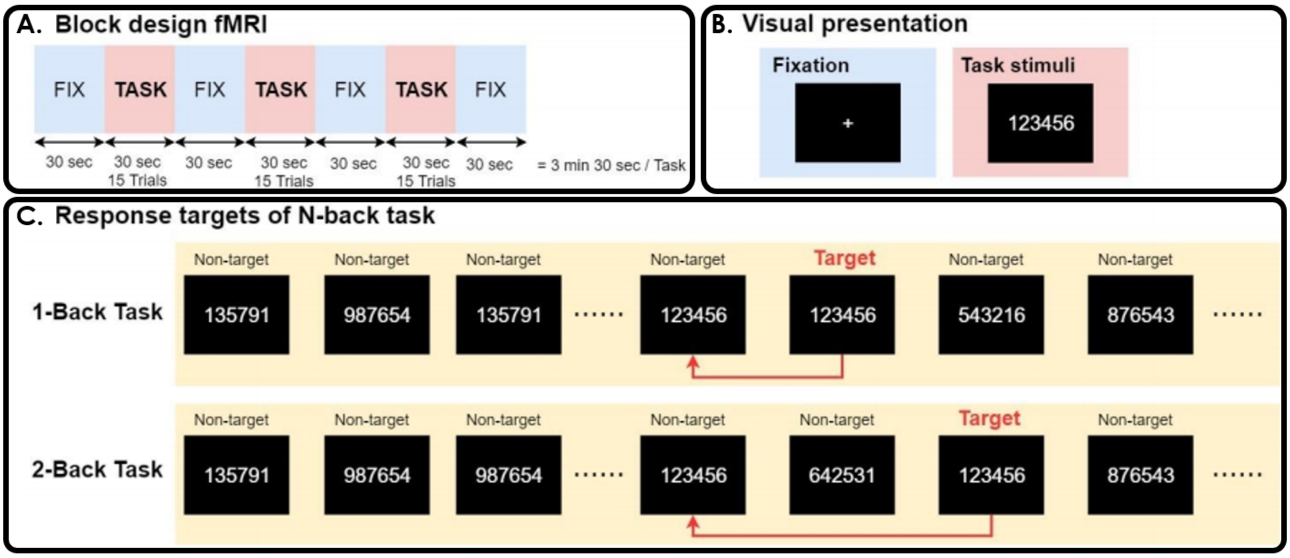

Figure S1. N-back WM task diagram.

(A) *N*-back task contains three epochs, (B) each composed of a 30-second task period and a 30-second fixing on a crosshair. The interstimulus interval between each trial during a task period was 2 s. In total, 45 trials were conducted, with each task consisting of 80% nontarget trials and 20% target trials. (C) In each run of the *N*-back tasks, participants were instructed to pay attention to a series of six-digit numerical stimuli and respond when the presented number matched the number presented *N* items before ($N = 1$ or 2).

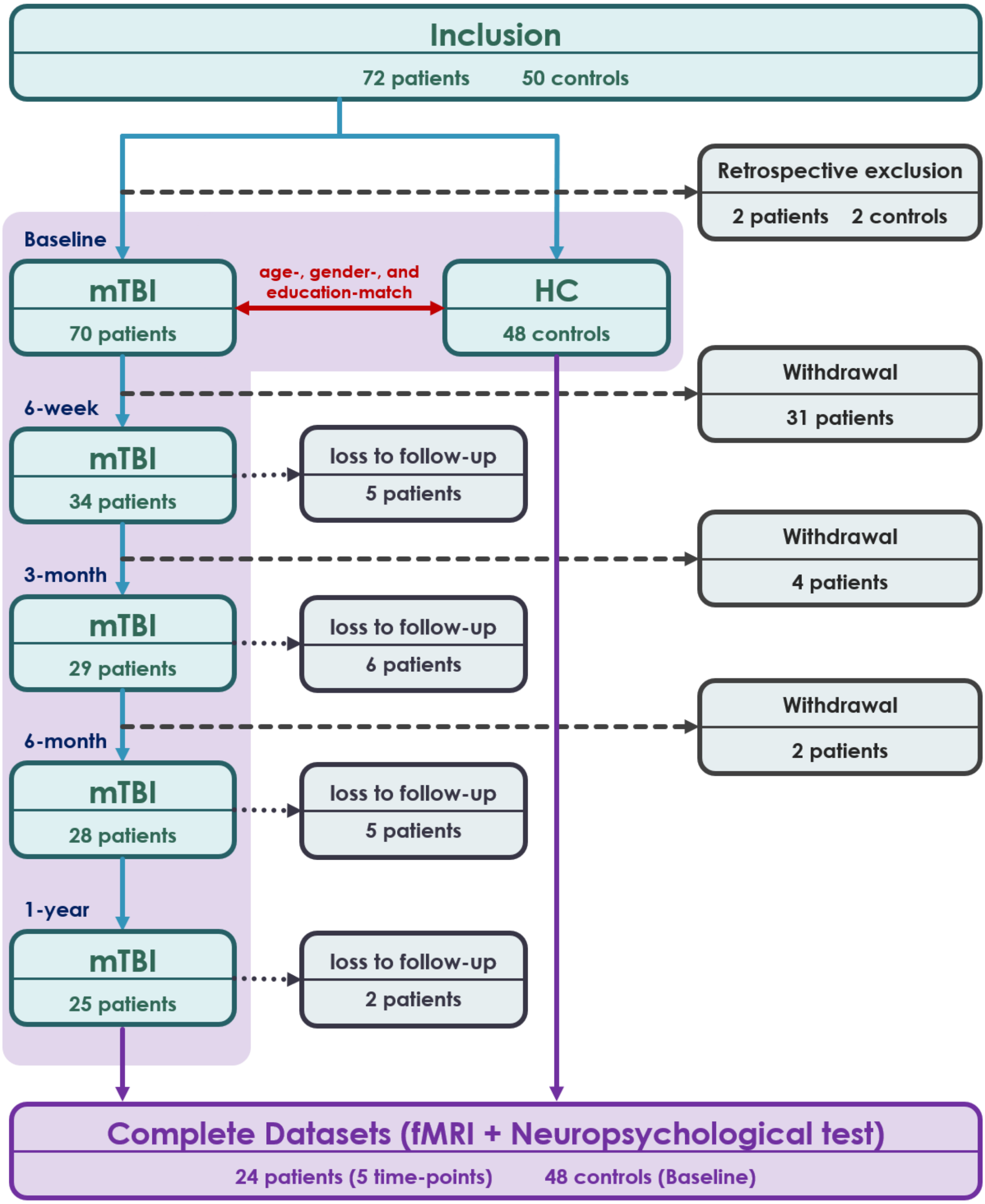

Figure S2. Flow chart.

Flow chart of participant inclusion and exclusion in this study.

Table S1. WM 2-back task-induced activation and deactivation peak regions among all participants

	Brain Regions	Side	MNI			Area	t value	
			X	Y	Z	(mm^3)	HC	mTBI
Basal Gangila	Caudate	L	-15	-1.5	16.5	2207	11.15	9.92
	Caudate	R	15	-1.5	16.5	2707	10.44	11.19
	Putamen	L	-19.5	6	12	857	5.56	5.89
	Putamen	R	19.5	6	12	780	6.61	7.76
Cerebellum	CRB crus1	L	-7.5	-78	-25.5	5798	12.94	9.65
	CRB crus1	R	6	-75	-28.5	2953	12.20	9.34
	CRB6	L	-28.5	-63	-25.5	10419	13.27	9.85
	CRB6	R	7.5	-75	-21	11684	15.09	8.65
	CRB4.5	L	-4.5	-61.5	-16.5	881	6.52	4.49
	CRB4.5	R	12	-57	-16.5	1310	6.71	4.42
	CRB10	L	-25.5	-31.5	-42	874	6.20	0.87
	CRB10	R	22.5	-36	-45	888	6.34	3.53
Task Positive Network	dlPFC	R	37.5	55.5	12	10625	7.35	5.24
	dlPFC	L	-37.5	55.5	12	7277	7.52	2.94
	FEF	R	31.5	6	60	8289	11.54	8.79
	FEF	L	-31.5	6	60	6311	9.50	6.01
	IFG(orb.)	R	48	12	33	6483	14.43	8.96
	IFG(orb.)	L	-39	7.5	30	3260	13.80	10.53
	IFG(tri.)	R	46.5	43.5	21	17135	6.18	3.66
	IFG(tri.)	L	-46.5	30	31.5	30463	7.77	4.86
	SPL	L	-25.5	-61.5	43.5	8127	15.13	12.81
	SPL	R	34.5	-58.5	51	6318	13.67	10.25
	IPS	L	-36	-51	45	12572	15.31	10.62
	IPS	R	36	-51	45	8451	17.08	12.14
	TPJ	L	-51	-40.5	21	2028	5.14	5.18
	TPJ	R	60	-34.5	21	2565	5.06	2.26
	rolandicoper.	L	-48	9	1.5	479	6.44	2.22
	rolandicoper.	R	51	7.5	16.5	284	6.68	6.16
	ITG	L	-45	-64.5	-9	5599	7.79	6.10
	ITG	R	51	-57	-19.5	9619	3.20	1.21
	Calcarine	L	-13.5	-90	-6	4023	14.73	8.62
	Calcarine	R	13.5	-90	-3	2440	7.51	8.21
	SMA	L	-3	15	52.5	10689	16.89	11.41
	SMA	R	1.5	9	61.5	8846	10.33	9.41
	dACC	L	-1	36	30	854	5.76	4.71
	dACC	R	7.5	25.5	30	2393	11.88	6.62
	AINS	L	-30	22.5	4.5	5073	16.62	13.93
	AINS	R	31.5	24	3	4330	19.50	14.85
Task Negative Network	PINS	L	-36	-19.5	19.5	1397	-9.33	-8.42
	PINS	R	37.5	-12	19.5	2612	-12.10	-9.92
	amyg.	L	-21	-6	-16.5	668	-6.14	-4.46
	amyg.	R	27	-1.5	-19.5	419	-4.24	-4.40
	MCC	L	-7.5	-28.5	43.5	6173	-8.20	-7.37
	MCC	R	12	-22.5	48	4577	-6.39	-5.06
	PCC	L	-4.5	-49.5	30	2514	-13.33	-13.06
	PCC	R	6	-48	30	1407	-13.27	-10.56
	aPFC	L	1.5	51	-7.5	3871	-7.66	-7.00
	aPFC	R	-6	46.5	-10.5	9430	-6.88	-5.48
	rmPFC	L	1.5	40.5	-15	5123	-11.39	-6.71
	rmPFC	R	-3	48	-10.5	7239	-10.51	-6.60
	vmPFC	L	1.5	19.5	-18	1299	-5.05	-0.18
	vmPFC	R	-3	24	-6	1775	-5.48	-3.43
	angular	L	46.5	-76.5	28.5	4475	-2.49	-1.45
	angular	R	-46.5	-72	28.5	7911	-8.01	-7.22
	IFC	L	-28.5	3.5	-10.5	2477	-6.40	-0.31
	IFC	R	19.5	22.5	-19.5	827	-3.18	-0.68
	MTG	L	-45	-67.5	24	6406	-7.63	-7.47
	MTG	R	48	-75	24	3898	-3.69	-2.45
	Hippo.	L	-24	-18	-18	4860	-8.06	-6.30
	Hippo.	R	27	-18	-18	3054	-8.35	-5.64

cerebellum; dACC = dorsal anterior cingulate cortex; dlPFC = dorsolateral prefrontal cortex; FEF = frontal eye fields; Hippo. = hippocampus; IFC = inferior frontal cortex; IFG = inferior frontal gyrus; IPS = inferior parietal sulcus; ITG = inferior temporal gyrus; L = left; MCC = middle cingulate cortex; MTG = middle temporal gyrus; Orb. = orbitalis; PCC = posterior cingulate cortex; R = right; rmPFC = rostro-medial prefrontal cortex; Rolandicoper. = Rolandic operculum; SMA = supplementary motor area; SPL = superior parietal lobe; TPJ = temporal parietal junction; Tri. = triangularis; vmPFC = ventromedial prefrontal cortex; fMRI = functional magnetic resonance imaging; mTBI = mild traumatic brain injury; WM = working memory; MRI = magnetic resonance imaging; PCS = postconcussive symptoms; HC = healthy control; MMSE = Mini-Mental State Examination; WAIS-IV = Wechsler Adult Intelligence Scale, fourth edition; WMI = WM index; AMT = arithmetic ability; DS = digit span score; SPM = Statistical Parametric Mapping; ROI = region-of-interest; SVM = support vector machine; ROC-AUC = area under receiver operating characteristic curve; DMN = default mode network.

Table S2. Demographics, clinical and cognitive characteristics, and WM task performance of the participants

		HC	mTBI	mTBI (6w)	mTBI (3m)	mTBI (6m)	mTBI (1Y)
**Sample size**		N = 48	N = 70		N = 24		
**Sex (female/male)**		32 / 16	47 / 23		16 / 8		
**Age**		37.44 ± 12.01	37.94 ± 12.24		33.29 ± 9.56		
**Education (year)**		14.36 ± 4.41	14.45 ± 2.96		15.14 ± 2.20		
**GOSE**		N.A.	6.20 ± 1.98		5.85 ± 2.18		
**Clinical symptoms**	**MMSE**	29.40 ± 0.76	28.44 ± 1.61 ***	29.05 ± 1.36	28.86 ± 1.46	28.90 ± 1.45	28.80 ± 1.45 *
	**PSQI**	4.90 ± 2.35	8.07 ± 4.36 ***	7.38 ± 4.40 **	7.29 ± 5.43 **	6.10 ± 4.09	6.67 ± 9.45 *
	**ESS**	5.63 ± 3.27	7.44 ± 4.23 *	6.89 ± 4.11	7.11 ± 4.23	5.85 ± 4.07	5.63 ± 3.32 †
	**DHI**	5.75 ± 7.17	18.64 ± 22.46 ***	12.10 ± 16.90 *	6.32 ± 11.97 †	10.48 ± 15.78	11.53 ± 17.35 †
	**PCSQ**	4.92 ± 5.73	13.30 ± 13.43 ***	8.24 ± 10.72	8.30 ± 10.77	4.33 ± 6.60 ††	9.03 ± 9.45 *
	**BAI**	4.33 ± 4.80	9.80 ± 9.57 ***	6.25 ± 8.32	4.95 ± 5.46 †	3.90 ± 5.49 ††	7.27 ± 7.84 *
	**BDI**	4.69 ± 4.95	9.70 ± 9.86 **	5.40 ± 7.05	6.63 ± 5.60	4.10 ± 5.35 †	6.97 ± 6.18 *
**WAIS - IV**	**DS**	34.28 ± 5.67	34.29 ± 6.50	34.40 ± 4.81	35.42 ± 5.02	34.08 ± 5.13	35.71 ± 5.27
	**Arithmetic**	16.63 ± 3.41	14.63 ± 4.40 **	15.31 ± 3.52 *	16.33 ± 3.43 ††	15.21 ± 4.00 *	15.52 ± 4.80 *
	**WMI**	109.72 ± 12.75	104.54 ± 17.33 **	105.60 ± 12.53 *	108.81 ± 12.49 †	105.02 ± 14.07 *	108.63 ± 14.28 *
	**PSI**	108.86 ± 12.24	95.92 ± 16.77 **	104.42 ± 16.61 ††	110.69 ± 18.22 †††	111.67 ± 16.85 †††	112.94 ± 16.72 †††
**WM task**	**1b ACC (%)**	95.37 ± 8.52	90.95 ± 8.09	97.88 ± 10.57 †	91.11 ± 10.57	98.94 ± 3.34 ††	95.79 ± 8.09
	**1b RT (ms)**	566.21 ± 114.73	577.31 ± 161.00	630.58 ± 107.52 *	617.13 ± 107.52	601.49 ± 111.80	609.08 ± 77.04 *†
	**2b ACC (%)**	71.53 ± 21.49	70.63 ± 26.08	73.54 ± 22.05	73.33 ± 22.05	76.72 ± 16.06	67.78 ± 20.14
	**2b RT (ms)**	717.14 ± 184.38	783.45 ± 187.51	715.80 ± 211.13	786.17 ± 211.13 †	757.35 ± 123.77	738.39 ± 101.81 †

Significance:

* indicated the significant difference compared with HC group. (*: $p < 0.05$, **: $p < 0.01$, ***: $p < 0.001$, FDR corrected)

† indicated the significant difference compared with the initial mTBI group. (†: $p < 0.05$, ††: $p < 0.01$, †††: $p < 0.001$, FDR corrected)

Abbreviations:

GOSE: Glasgow Outome Scale-Extended
MMSE: Mini-Mental State Examination
PSQI: Pittsburgh Sleep Quality Index
ESS: Epworth Sleepness Scale
DHI: Dizziness Handicap Inventory
PCSQ: Post-Concussive Symptoms Questionnaire
BAI: Beck Anxiety Inventory
BDI: Beck Depression Inventory
WAIS: Wechsler Adult Intelligence Scale
DS: Digit Span
WMI: Working Memory Index
PSI: Processing Speed Index
WM: Working Memory
ACC: Accuracy
RT: Reaction Time
FDR: False Discovery Rate
